# A monoclonal antibody against lymphocyte function-associated antigen-1 decreases HIV-1 replication by inducing the secretion of an antiviral soluble factor

**DOI:** 10.1186/1743-422X-10-120

**Published:** 2013-04-18

**Authors:** Jenna Rychert, Lindsay Jones, Graham McGrath, Sue Bazner, Eric S Rosenberg

**Affiliations:** 1 Departments of Medicine and Pathology, Massachusetts General Hospital and Harvard Medical School, Boston, MA, USA

**Keywords:** HIV-1, LFA-1, Monoclonal antibody therapy

## Abstract

**Background:**

Lymphocyte Function-Associated Antigen-1 (LFA-1) likely plays a role in the pathogenesis of against HIV-1 and is known to facilitate cell-to-cell transmission of the virus. A monoclonal antibody specific for LFA-1 (Cytolin®) was evaluated as a potential therapeutic in pilot studies performed in the mid-1990s. These uncontrolled human studies suggested that administration of this anti-LFA-1 antibody to HIV-1 infected individuals could provide a modest benefit by decreasing circulating HIV-1 RNA and increasing CD4+ T cell counts. At the time, it was proposed that when bound to cytolytic T cells, the antibody inhibited lysis of activated CD4+ T cells. Given the renewed interest in monoclonal antibody therapy for HIV-1 infected individuals, we investigated possible mechanisms of action of this antibody *in vitro*.

**Methods:**

To assess whether this anti-LFA-1 antibody binds to HIV-1, a virus capture assay was performed. Binding of the antibody to cells was assessed using flow cytometry. Inhibition of HIV-1 replication was determined in culture by measuring the amount of p24 produced by ELISA. After co-culture of the antibody with peripheral blood mononuclear cells, supernatants were assayed for cytokines and chemokines using various immunoassays.

**Results:**

Our experiments demonstrate that anti-LFA-1 antibody binds to CCR5 and CXCR4 utilizing strains of HIV-1. It also binds to CD8+ T cells and dendritic cells. When bound to virus prior to infection, there is no decrease in HIV-1 replication, suggesting it does not directly inhibit viral replication via virus binding. When bound to cells, it does not inhibit lysis of CD4+ T cells, as was originally hypothesized. Binding to cells does appear to induce the production of a soluble factor that inhibits HIV-1 replication. We determined that this soluble factor was not any of the cytokines or chemokines with known anti-HIV-1 activity. Further, the antibody does not appear to induce any common immune modulating cytokines or chemokines.

**Conclusions:**

These results suggest that one possible mechanism of action of this anti-LFA-1 antibody is to inhibit HIV-1 replication via the production of a soluble antiviral factor that is induced upon binding to cells.

## Background

Lymphocyte Function-Associated Antigen-1 (LFA-1, CD11a/CD18) is a member of the integrin family of adhesion molecules. It is expressed on immune cells and plays a role in leukocyte trafficking, antigen presentation, cellular activation, and adhesion of Cytotoxic T lymphocytes (CTL) to their targets. In addition to its role in the immune response, LFA-1 and its ligands are incorporated into the viral envelope as HIV-1 buds from the cell surface [1]. These proteins facilitate viral synapse formation and promote cell-to-cell transmission of the virus [2].

Cytolin® is a murine anti-human monoclonal antibody that binds to LFA-1 (hereafter referred to as LFA-1 MAb). It recognizes an epitope within CD11a known as S6F1. This epitope is preferentially expressed on CD8+ T cells and can be used to distinguish killer effector cells from suppressor effector cells [3]. In HIV-1 infected individuals, CD8+ T cells play a central role in controlling viral replication by lysing infected cells. It has been shown that the frequency of CD8+ T cells expressing the S6F1 epitope is higher in HIV-1 infected individuals compared to uninfected controls [4,5]. The S6F1+ cell subset is not expanded in individuals with Epstein Barr Virus (EBV) infection [6], suggesting this epitope may play a unique role in the pathogenesis of HIV-1 infection and is not merely the result of expansion of this cell subset due to chronic viral infection.

It has been hypothesized that LFA-1 MAb could be used therapeutically to alter the course of HIV-1 infection. In several small clinical studies [7,8] a reduction in HIV-1 RNA (range 0.2-1 log_10_ copies/ml) and a modest increase in CD4 T cell count (range 70–200 cells/mm^3^) was observed when the antibody was administered to HIV-1 infected individuals. At the time, investigators hypothesized that LFA-1 MAb improved CD4 T cell counts by inhibiting the cytolytic effect of CD8+ T cells (CTL) on activated uninfected CD4+ T cells [7]. This hypothesis was based on *in vitro* studies that suggested that CTL killing of activated uninfected CD4+ T cells contributes to CD4+ T cell depletion in HIV-1 infected individuals [9,10]. This lytic activity was shown to be abrogated using an antibody specific to LFA-1 [10]. Given the increased interest in monoclonal antibody therapy in persons with HIV-1 infection, we set out to determine possible mechanisms of action of LFA-1 MAb.

## Results

### Patient characteristics

We recruited a cohort of individuals for blood donation including 12 HIV-1 positive and 13 HIV-1 negative subjects. Blood was collected at multiple time points from each study subject over a 21-month period. The demographics and clinical characteristics of the cohort are summarized in Table 1. The HIV-1 infected subjects in the cohort were in the asymptomatic phase of infection and were not on antiretroviral therapy. The mean HIV-1 plasma RNA viral load was 18,495 copies/ml and was similar over the course of the study (p = 0.84, repeated measures ANOVA). The mean CD4 T cell count in HIV-1 infected subjects was 661 cells/mm^3^, which was slightly lower than the HIV-1 negative subjects (mean = 859 cell/mm3) and remained relatively unchanged (p = 0.76, repeated measures ANOVA).

**Table 1:** Cohort characteristics

**Cohort demographics**	**n = 25**		
Male	72%		
Female	28%		
Caucasian	72%		
African American	12%		
Asian	16%		
Mean Age (years)	36		
**Clinical Parameters**	**HIV**	**HIV**	
	**Negative**	**Positive**	
	**(n = 13)**	**(n = 12)**	
Mean HIV Plasma Viral Load at first visit copies/ml (range)	ND	18,495	
		(289–85,500)	
Mean Absolute CD4 Count at first visit cells/mm^3^ (range)	859	661	NS
	(415–1624)	(385–1150)	
Mean Absolute CD8 Count at first visit cells/mm^3^ (range)	478	1153	p < 0.0001
	(231–766)	(695–2722)	

*NS*, not significant.

*ND*, not detected.

### Inhibiting HIV-1 replication via virus binding

**Figure 1:**
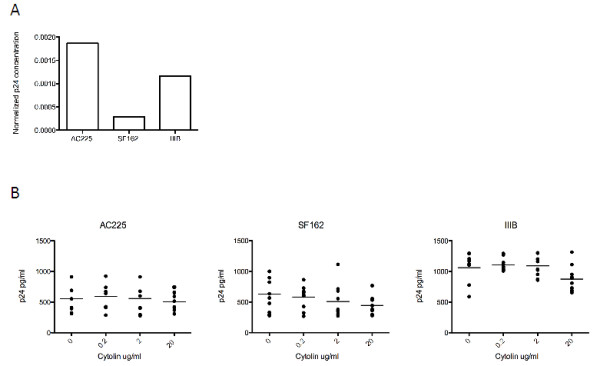
**LFA-1 MAb does not directly inhibit HIV replication via binding to virions.** (**A**) A virus capture assay was used to determine whether LFA-1 MAb binds to HIV-1. Triplicate wells were coated with 5 μg/ml of LFA-1 MAb or PBS as a control. Undiluted virus stocks were then added to each well and the presence of bound virus was determined using a p24 ELISA. Results are expressed as the concentration of captured p24 divided by the concentration of p24 in the undiluted virus stock. (**B**) PHA-activated CD8+ cell depleted PBMC from 10 HIV-1 negative subjects were infected in triplicate with the same three HIV-1 isolates in the presence of increasing concentrations of LFA-1 MAb. The degree of HIV-1 replication was determined using a p24 ELISA on the supernatant at day 7. Each dot represents a single subject. The line represents the mean for each concentration of LFA-1 MAb tested. There was no significant difference in replication in the presence of LFA-1 MAb as compared to PBS only control for any of the viruses tested.

Given that LFA-1 can be incorporated into the HIV-1 envelope upon budding, we asked whether LFA-1 MAb could inhibit HIV-1 replication via binding to virus. To address this, we first performed a virus capture assay to determine whether LFA-1 MAb could bind to HIV-1. Undiluted virus stocks representing both CCR5 utilizing (HIV-SF162, HIV-AC225) and CXCR4 utilizing (HIV-IIIB) HIV-1 strains were incubated in wells coated with LFA-1 MAb. Captured virions were then detected using a p24 ELISA. HIV-1 p24 was not detected in control wells containing PBS. The mean concentration of wells containing LFA-1 MAb was 112 pg/ml for HIV-AC225, 43 pg/ml for HIV-SF162, and 127 pg/ml for HIV-IIIB. To determine whether LFA-1 MAb bound one isolate better than another, we normalized this data to the p24 content of each viral stock (AC225 60,040 pg/ml, SF162 148,986 pg/ml, IIIB 108,727 pg/ml). As shown in Figure 1A, LFA-1 MAb captured the primary isolate, HIV-AC225 better than the other CCR5 utilizing isolate, HIV-SF162, and about the same as the CXCR4 utilizing isolate, HIV-IIIB. To better interpret these data, we performed a similar assay, in parallel, using a mixture of monoclonal antibodies specific to HIV-gp120 to capture each viral stock rather than LFA-1 MAb. The gp120-specific antibodies were slightly better at capturing all three viral isolates. In this case, the amount of p24 captured was 270 pg/ml for HIV-AC225, 87 pg/ml for HIV-SF162, and 262 pg/ml for HIV-IIIB. Given that the virus specific antibodies (anti-gp120) were able to capture a similar amount of virus as the LFA-1 MAb, we conclude that LFA-1 MAb binds to virus particles.

We then asked whether HIV-1 replication could be inhibited in the presence of LFA-1 MAb. The same viral stocks were incubated with increasing concentrations of LFA-1 MAb prior to infecting PHA activated CD8-depleted PBMC. These infection assays were performed in triplicate using cells from ten of the HIV-1 negative subjects. As shown in Figure 1B, there was a trend for a decrease in replication with higher concentrations of LFA-1 MAb for HIV-IIIB and to a lesser extent HIV-SF162. However, the mean concentration of p24 was not significantly different when virus was pre-incubated with LFA-1 MAb for any of the three viral isolates (p = 0.07 for IIIB, p = 0.55 for SF162, p = 0.56 for AC225, one way ANOVA). Although LFA-1 MAb is able to bind to CXCR4 and CCR5 utilizing strains of HIV-1, this does not result in the inhibition of viral replication.

### Inhibiting HIV replication via cell binding

**Figure 2:**
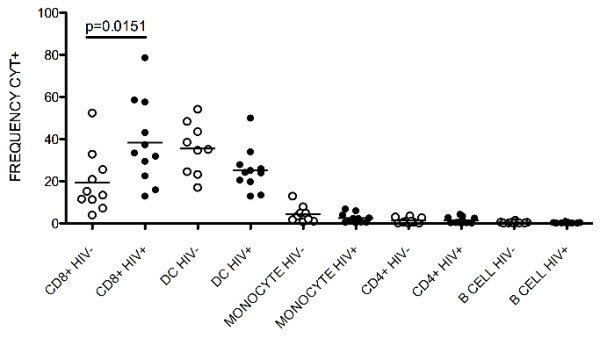
**LFA-1 MAb binds to CD8+ T cells and dendritic cells.** PBMC from 10 HIV-1 negative (open circles) and 11 HIV-1 positive subjects (closed circles) were incubated with LFA-1 MAb and fluorochrome labeled antibodies that differentiated immune cell subsets then analyzed using flow cytometry. Results are expressed as the percent of LFA-1 MAb positive cells within each cell subset for each subject tested. The horizontal line indicates the mean percent within each subset. The mean percent of CD8+ LFA-1 MAb positive cells was significantly higher in HIV infected subjects (p = 0.0151, Mann Whitney test). There was no significant difference in the percent of LFA-1 MAb positive cells in the remaining cell subsets.

The S6F1 epitope, to which LFA-1 MAb binds, was previously shown to be preferentially expressed on CD8+ T cells [3]. Flow cytometric analysis of PBMC was used to verify this finding and identify other cell subsets that LFA-1 MAb binds. PBMC from 10 of the HIV-1 negative and 11 of the HIV-1 positive subjects were incubated with LFA-1 MAb and antibodies that differentiate T cells, B cells, dendritic cells, and monocytes. The percent of LFA-1 MAb positive cells was highest in the CD8+ T cell subset (mean = 19.43 for HIV-1 negative, mean = 38.31 for HIV-1 positive), followed by dendritic cells (mean = 35.46 for HIV-1 negative, mean = 25.24 for HIV-1 positive). We identified very few monocytes (mean = 4.4 for HIV-1 negative, mean = 2.5 for HIV-1 positive), CD4+ T cells (mean = 1.2 for HIV-1 negative, mean = 1.4 for HIV-1 positive), or B cells (mean = 0.4 for HIV-1 negative, mean = 0.3 for HIV-1 positive) that bound LFA-1 MAb (Figure 2). The percent of CD8+ T cells that bound LFA-1 MAb was significantly higher in HIV-1 positive subjects (p = 0.0151, Mann Whitney test) and correlated with the absolute CD8+ T cell count (p = 0.0011, Pearson correlation, data not shown). HIV-1 negative and HIV-1 positive subjects had similar percentages of LFA-1 MAb positive cells in the remaining cell subsets (p = 0.08 for dendritic cells, p = 0.68 for CD4+ T cells, p = 0.22 for monocytes, p = 0.53 for B cells; Mann Whitney test). Given that the conformation of LFA-1 can change based on the activation status of a cell, we performed a similar analysis on cells activated with PMA and Ionomycin prior to cell surface staining. We found no difference in the mean frequency of LFA-1 MAb positive cells for any of these cell subsets when inactivated and activated cells were compared (p = 0.82 for CD8+ T cells, p = 0.62 for dendritic cells, p = 0.53 for monocytes, p = 0.71 for CD4+ T cells; Mann Whitney test; data not shown).

**Figure 3:**
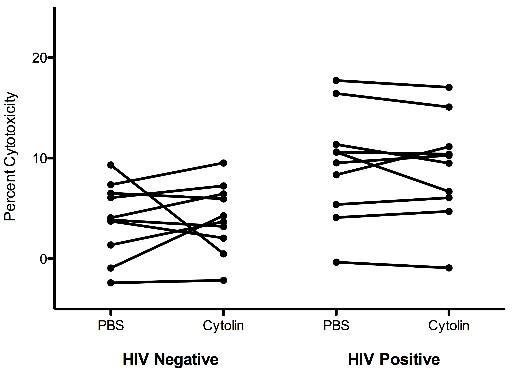
**LFA-1 MAb does not inhibit CTL killing of activated uninfected CD4+ target cells.** CD4-depleted PBMC effector cells from ten HIV-1 negative and ten HIV positive subjects were co-cultured at a ratio of 25:1 with activated uninfected CFSE labeled CD4+ target cells and cytotoxicity was determined using flow cytometry to differentiate 7AAD + (dead) and 7AAD- (live) target cells. Effector cells from HIV positive subjects were more cytotoxic (p = 0.0115 for PBS, p = 0.0185 for LFA-1 MAb, Mann Whitney test) but the degree of cytotoxicity did not decrease significantly in the presence of LFA-1 MAb for cells from either HIV-1 positive or HIV-1 negative subjects (p = 0.879 for HIV-1 negative, p = 0.5286 for HIV-1 positive, paired *t* test).

It was initially proposed that LFA-1 MAb could prevent the loss of CD4 T cells in HIV-1 infected individuals by inhibiting CTL from lysing activated uninfected CD4+ T cells [7]. To test this hypothesis, activated uninfected CD4+ target cells were co-cultured with CD4-depleted PBMC effector cells in the presence and absence of LFA-1 MAb and the amount of cytotoxicity was determined by flow cytometry. In this assay, target cells are stained with CFSE to differentiate them from effector cells and all cells are stained with 7AAD, a viability dye that specifically stains dead cells. This allows dead target cells (CFSE + 7AAD + cells) to be readily enumerated. Effector cells from HIV-1 positive subjects were more cytotoxic than effector cells from HIV-1 negative subjects, regardless of whether they were incubated with PBS (p = 0.0115, Mann Whitney test) or LFA-1 MAb (p = 0.0185 LFA-1 MAb, Mann Whitney test) (Figure 3). In the presence of LFA-1 MAb, the frequency of dead target cells decreased in cultures from 4 of 10 HIV-1 negative and 5 of 10 HIV-1 positive subjects. However, the mean percent cytotoxicity was similar in the presence or absence of LFA-1 MAb for both groups (p = 0.879 for HIV-1 negative, p = 0.5286 for HIV-1 positive, paired *t* test). These data suggests that LFA-1 MAb does not inhibit CTL killing of activated uninfected CD4+ T cells. Thus, we were unable to substantiate the previously hypothesized mechanism of action [7].

**Figure 4:**
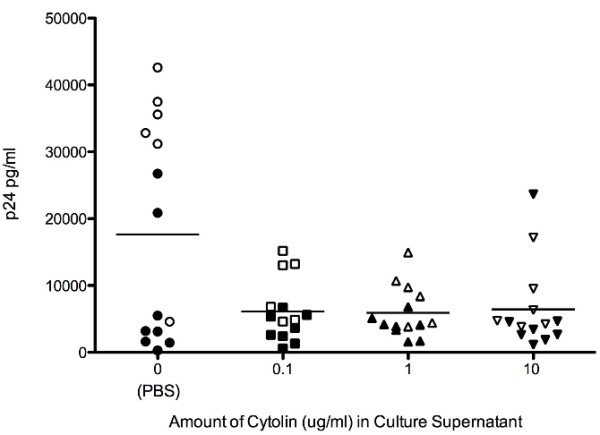
**LFA-1 MAb induces the production of an anti-viral soluble factor.** PBMC from six HIV negative (open symbols) and eight HIV-1 positive subjects (closed symbols) were incubated for 24 hours in the presence of increasing concentrations of LFA-1 MAb or PBS alone (x-axis). Supernatant was harvested from these cultures then mixed with fresh CD8-depleted PBMC and subsequently infected with 10 TCID50 of HIV-SF162. The degree of viral replication was measured on day 7 using a p24 ELISA. There was a significant decrease in the mean concentration of p24 (indicated by the horizontal line) in cultures containing LFA-1 MAb (p = 0.0006, one way ANOVA).

LFA-1 can act as both an adhesion and a signaling protein [11]. The integrin family to which LFA-1 belongs has been shown to transmit signals when bound by ligand, resulting in the production of inflammatory cytokines [12]. Given that HIV-1 replication is inhibited by several cytokines and chemokines, we next asked whether, when bound to cells, LFA-1 MAb induced the production of a soluble factor that would inhibit HIV-1 replication. To evaluate this question, PBMC from eight of the HIV-1 positive and six of the HIV-1 negative donors were incubated with increasing concentrations of LFA-1 MAb for 24 hours. The supernatant from these cultures was then added to a viral replication assay and the degree of HIV-1 replication was assessed by measuring the production of p24 antigen (Figure 4). As a positive control, culture media was used in place of supernatant to ensure a productive infection (p24 = 17,157 pg/ml). There was a significant decrease in HIV-1 replication in the presence of supernatants stimulated by LFA-1 MAb compared to supernatants stimulated with PBS alone (p = 0.0006, one way ANOVA). Supernatants from five HIV-1 negative and two HIV-1 positive subjects inhibited replication by greater than 85% at every concentration of LFA-1 MAb tested. From these data we conclude that LFA-1 MAb can induce the production of a soluble antiviral factor. In the seven other subjects, PBS stimulated control supernatants inhibited HIV-1 replication and no further inhibition was observed when LFA-1 MAb stimulated supernatants from these subjects were tested, suggesting that cells from some individuals produce antiviral factors spontaneously and LFA-1 MAb does not further enhance this production.

### Identifying the antiviral soluble factor

**Figure 5:**
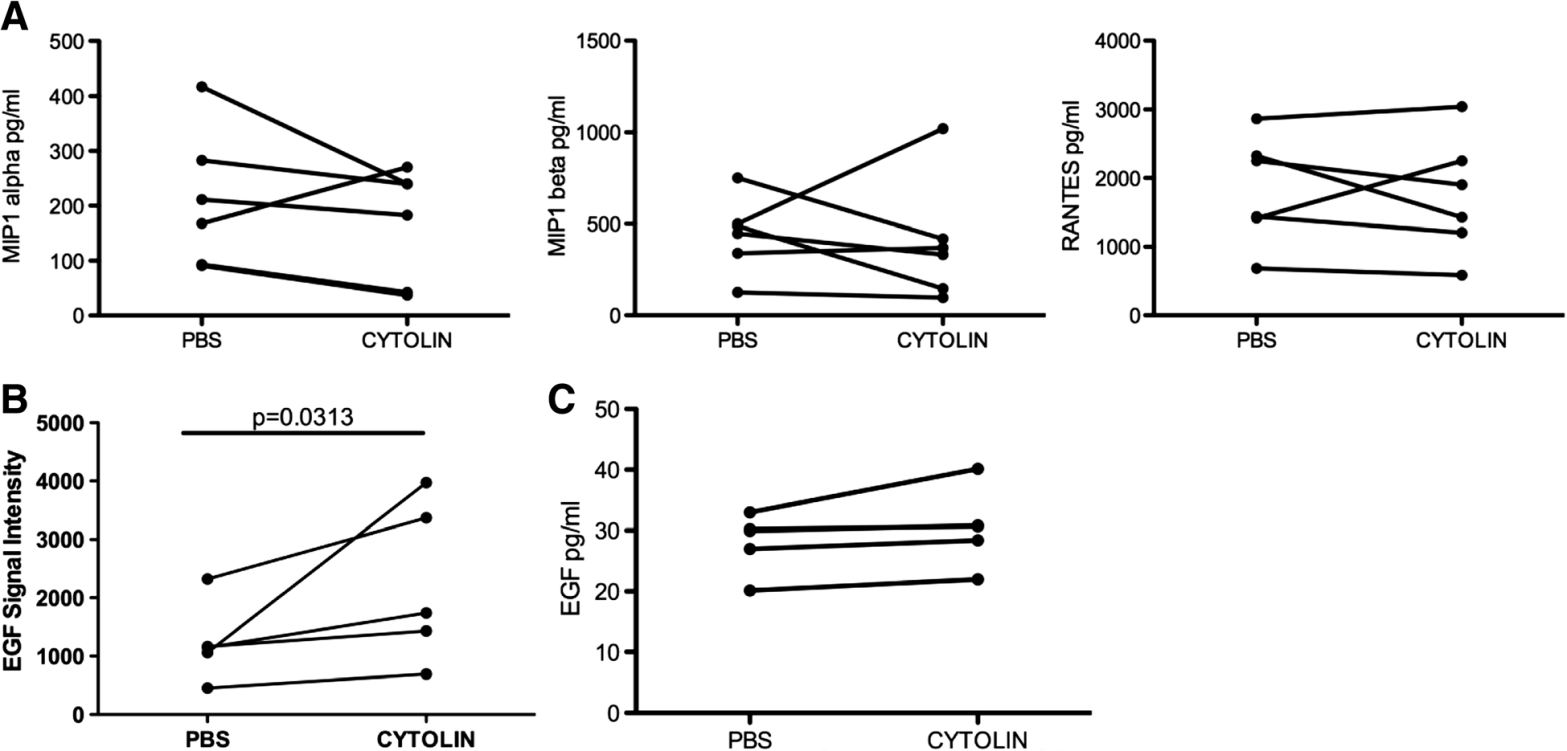
**Search for the identity of the anti-viral soluble factor.** (**A**) Supernatants with anti-viral activity, obtained from culturing PBMC in the presence of LFA-1 MAb, were tested for MIP1-alpha, MIP1-beta, and RANTES using a bead based ELISA assay. Results are expressed as the concentration of each analyte in paired control and LFA-1 MAb treated supernatants. (**B**) Supernatants were then tested for the presence of 174 different analytes using a fluorescence based antibody array. Results from this semi-quantitative assay are expressed in relative light units. Epidermal Growth Factor (EGF) was the only analyte where the signal intensity differed significantly between LFA-1 MAb treated and control supernatants (p = 0.0313). (**C**) To confirm this finding, the concentration of EGF was then determined by standard quantitative ELISA. Unless noted, the difference between LFA-1 MAb and control treated supernatants were not significant.

Given that PBMC produced the antiviral soluble factor within 24 hours, we hypothesized that the factor may be one of the cytokines or chemokines with known anti-HIV-1 activity [13-15]. We looked for analytes that were present at higher concentrations in the LFA-1 MAb treated supernatants than in the PBS treated supernatants. Our first set of candidates included the beta chemokines, MIP1-alpha, MIP1-beta, and RANTES. As shown in Figure 5A, there was no significant increase in any of these chemokines in the presence of LFA-1 MAb (p = 0.16 for MIP1-alpha, p = 0.34 for MIP1-beta, p = 0.28 for RANTES, Wilcoxon sign rank test). We next tested the supernatants for alpha-defensins (HNP 1, 2, and 3) and Interferon-alpha (IFN-alpha). The concentration of these analytes in both the control supernatants and the LFA-1 MAb treated supernatants was below the limit of detection of the assays (data not shown). Finally, we broadened our search for the possible identity of the soluble factor using an antibody array. This array was used to test for the presence of 174 different analytes including SDF-1, MDC, LIF, MCP-2, Lymphotactin, IL-8, IL-10, IL-16, TGF-β and other common cytokines, chemokines, and growth factors (for full list of analytes see Additional file 1). Using this semi-quantitative strategy, we identified a single analyte where the mean signal intensity in LFA-1 MAb treated supernatants was higher than the mean signal intensity in PBS treated supernatants after subtracting out the signal intensity of the media control. As shown in Figure 5B, the mean signal intensity of Epidermal Growth Factor (EGF) in LFA-1 MAb treated supernatants was 2241 relative units and significantly higher than the mean signal intensity in PBS treated supernatants with a mean of 1230 relative units (p = 0.03, Wilcoxon sign rank test). Using a quantitative ELISA for EGF, we were unable to verify these results (Figure 5C). We, therefore, conclude that the soluble factor induced by incubation of cells with LFA-1 MAb is not one of the known HIV-1 inhibitory cytokines or chemokines. Furthermore, it is not one of the common cytokines and chemokines we tested for. This implies that LFA-1 MAb does not induce any common immunomodulating cytokines and chemokines.

## Discussion

In this study, we set out to determine the *in vitro* mechanisms of action of a monoclonal antibody that binds to Lymphocyte Function-Associated Antigen-1 (LFA-1). We found that LFA-1 MAb is able to bind HIV-1 virions and preferentially binds to CD8+ T cells and dendritic cells. When bound to virus, it does not inhibit HIV-1 replication in culture. When bound to cells, it does not inhibit CTL killing of activated uninfected CD4+ T cells, as was previously hypothesized; rather, it appears to induce the production of an as yet unidentified soluble factor that can inhibit HIV-1 replication.

Using a virus capture assay, we determined that LFA-1 MAb is able to bind to HIV-1. However, it does not appear to bind to CD4+ T cells. We know that LFA-1 takes on different conformations based on the activation state of a cell [16,17]. It is possible, that under specific conditions, infected CD4+ T cells express LFA-1 in such a way that LFA-1 MAb could bind. Indeed, others have shown that HIV-1 gp120 can trigger the activated state of LFA-1 in a CD4 dependent manner [18]. It has also been shown that binding of α4β7 by gp120 results in an altered conformation of LFA-1 on CD4+ T cells [19]. It is therefore possible that HIV-1 infection itself results in changes in the conformation of LFA-1 on CD4+ T cells such that they express the S6F1 epitope to which LFA-1 MAb binds. Alternatively, LFA-1 MAb may only bind to the virions that replicated within dendritic cells, which contain LFA-1 on their surface.

Incubating PBMC with LFA-1 MAb for 24 hours resulted in the production of a soluble factor that inhibited HIV-1 replication. Given the cells that LFA-1 MAb binds and the short period of incubation, we hypothesized that this factor was most likely a chemokine or cytokine. However, we did not observe an increase in the concentration of any of the analytes we tested in supernatants from LFA-1 MAb exposed PBMC, as compared to control. It is possible that a combination of these factors may explain the anti-HIV-1 activity we observed or that the antiviral soluble factor may vary from one culture to another. This is difficult to assess given the small number of cultures tested. Another possibility is that residual LFA-1 Mab in the supernatant contributed to the inhibition we observed. However, we did not observe inhibition of viral replication when LFA-1 MAb is incubated with virus prior to infection. It is also possible that this soluble factor is the same anti-HIV-1 soluble factor, known as “CD8 Antiviral Factor” (CAF). CAF is known to be produced by CD8+ T cells [20]; however, it is typically produced after stimulation with anti-CD3 antibodies and its production is maximized after 5–9 days of culture. Therefore, it seems unlikely that the soluble factor is CAF. Further studies to identify the soluble factor are warranted. Biochemical experiments to determine it’s approximate size, it’s sensitivity to proteases, and it’s tolerance to changes in pH and temperature may yield important clues to its identity; however, ultimately it may be necessary to isolate it from the complex mixture of analytes in the cell culture supernatant and produce it in sufficient quantities to get a definitive identification.

In this study, we have shown that LFA-1 MAb may be able to inhibit HIV-1 replication as a result of binding to either CD8+ T cells or dendritic cells and inducing the production of an antiviral soluble factor. This is just one of the potential mechanisms by which this antibody could decrease HIV-1 replication *in vivo*. Other potential mechanisms include interfering with cell-to-cell transmission of virus or augmenting the HIV-1 specific immune response by altering leukocyte trafficking. Additional studies are needed to examine these mechanisms in more detail.

Our approach to identifying the antiviral soluble factor does give us some insight into the impact that LFA-1 MAb may have on the immune system. Despite binding to a signaling molecule that has the potential for altering an immune response, we did not identify any chemokine or cytokine that was produced as a consequence of the interaction between LFA-1 MAb and LFA-1. Although not shown here, we also did not observe an increase in proliferation or apoptosis in cultures containing LFA-1 MAb. Thus, this antibody appears to have a benign effect, in general, on immune cells. This could be advantageous if this antibody is further tested in human trials.

## Conclusions

From this study we conclude that the mechanism of action of LFA-1 MAb is not the result of the antibody binding to virus, but rather a downstream effect of it binding to cells. Our data suggest that binding of LFA-1 MAb to CD8+ T cells or dendritic cells may result in the production of a yet-to-be-identified soluble antiviral factor.

## Methods

### Materials

Cytodyn Incorporated (Santa Fe, New Mexico) provided the anti-LFA-1 monoclonal antibody (Cytolin®). HIV-SF162 and HIV-IIIB were obtained from the NIH AIDS Reference and Reagent Program. These two viruses were chosen because they utilize either CCR5 or CXCR4 as a co-receptor for viral entry (SF162 and IIIB respectively). HIV-AC225 is a primary isolate from a recently infected individual. It is predicted to be a CCR5 utilizing isolate based on the sequence of the V3 region of its envelope. All three viruses were propagated on CD8-depleted PBMC from the same donor. Culture media was prepared using RPMI supplemented with Hepes, Penicillin/Streptomycin, L-glutamine, and 10% fetal calf serum.

### Subjects

Twenty-five subjects (13 HIV-1 negative and 12 HIV-1 positive) were enrolled in a blood draw only study. To be included in the study, HIV-1 infected subjects had to be in the asymptomatic stage of infection with a CD4 T cell count greater than 350 cells/mm^3^, an HIV-1 plasma RNA viral load less than 100,000 copies/ml, and not be on antiretroviral therapy. All subjects signed informed consent as approved by the Massachusetts General Hospital (MGH) Human Subjects Committee.

Peripheral blood samples were obtained by venipuncture into tubes containing Acid Citrate Dextrose (ACD). Plasma was separated from whole blood using centrifugation. Peripheral blood mononuclear cells (PBMC) were obtained by density gradient centrifugation (FICOLL, Sigma). HIV-1 serostatus was confirmed at the initial visit in all subjects. T cell subset analysis was performed at every visit for all subjects. HIV-1 RNA testing was performed at the initial visit for all subjects, and then only for HIV-1 infected subjects at subsequent visits. Assays were performed in the Clinical Laboratory at MGH.

### Virus capture

To determine whether LFA-1 MAb could bind to HIV-1, a virus capture assay was performed similar to what has previously been described [21-23]. Briefly, ninety-six well plates (Nunc) were coated with 5ug/ml of the anti-LFA-1 antibody, PBS, or 1 ug/ml mixture of monoclonal antibodies specific to HIV-1 gp120, for 2 hours at room temperature. The gp120-specific antibody mixture contained three human monoclonal antibodies, 17b, A32, and EH21. These antibodies bind to discontinuous epitopes, are known to cross-react with envelope glycoproteins from multiple Clade B isolates, and likely bind to monomeric gp120 [24-27]. After blocking with PBS containing 4% whey, undiluted virus stocks were plated in triplicate, and incubated at room temperature for 2 hours. Wells were washed with PBS containing 0.05% Tween20, and harvested with 100ul PBS containing 0.5% TritonX 100. This supernatant was kept at 4°C overnight then diluted to 1:500 in PBS containing lysing solution. The presence of virus in these supernatants was determined using a p24 ELISA, as per the manufacturers instructions (HIV-1 p24 ELISA, Zeptometrix).

### Isolation of cell subsets

PBMC were depleted of CD8+ cells by magnetic bead based separation using Dynabead CD8 (Invitrogen) as per the manufacturers instructions. This resulted in >95% depletion of CD8+ cells. CD4+ cells were isolated from PBMC by positive selection using paramagnetic beads coupled to anti-CD4 monoclonal antibodies (Dynabead CD4, Invitrogen). This isolation was performed according to the manufacturers instructions and resulted in a population of cells containing >95% CD4+ cells.

### CTL lysis of activated CD4+ T cells

To determine whether LFA-1 MAb abrogated CTL lysis of activated uninfected CD4+ cells, we first obtained CD4+ target cells using magnetic positive enrichment, as described above. These cells were activated for three days at 37°C with 1.25 ug/ml PHA (phytohemagglutinin) in media containing 50U/ml IL-2. Autologous CD4+ cell depleted PBMC were used as effector cells. These cells were incubated with 10 ug/ml of LFA-1 MAb or PBS, as a control, on ice for 30 minutes then added without washing to the target cells. The activated CD4+ target cells were differentiated from effector cells via staining with 1uM CFSE. Effector and target cells were combined at a ratio of 25:1. After a 4 hour co-culture at 37°C, all cells were stained with 1 ug/ml 7AAD for 20 minutes at room temperature. The cells were then washed in PBS containing 2 ug/ml Actinomycin D and 1% fetal calf serum. Prior to acquisition, the cells were fixed in Actinomycin D containing buffer with 1% formaldehyde. The frequency of CFSE + 7AAD + cells (dead targets) was determined by flow cytometry on a BD LSRII. Single stained controls were used to set the gates. Percent cytotoxicity was calculated using the following formula: 100*(%sample lysis-%target cell only lysis)/(100-% target cell only lysis).

### Identifying cells that bind LFA-1 MAb

To determine the cell subsets that bind to this anti-LFA-1 antibody, flow cytometry was performed. PBMC were either left inactivated or were activated for 1 hour at 37°C with 10 ng/ml of PMA (phorbol 12-myristate 13-acetate) and 5 ug/ml Ionomycin. The cells were then incubated with 24 ug/ml of LFA-1 MAb for 20 minutes at 4°C. This concentration of anti-LFA-1 was determined by titration on PBMC using flow cytometry. After washing in PBS containing 1% fetal calf serum, cells were stained with a FITC-labeled anti-mouse IgG for 20 minutes at 4°C. The various cell subsets were identified using fluorochrome-labeled antibodies to discriminate T cells, B cells, monocytes, and dendritic cells (CD3 PE, CD4 Q605, CD8 APC-Cy7, CD14 Pacific Blue, CD19 PE-Cy5, CD11c APC). The cells were washed and fixed in 1% formaldehyde prior to acquisition on a BD LSR-II. Gating was performed manually to identify CD3+ CD19- T cells, CD19 + CD3- B cells, and CD19-CD3- monocytes and dendritic cells. The T cell subset was further differentiated into CD4+ and CD8+ cells. Dendritic cells were defined as CD14- CD11c+, while monocytes were defined as CD14+ CD11c+. The percent of LFA-1 MAb + cells within each cell subset is reported.

### Induction of soluble antiviral factors

To induce the production of an antiviral soluble factor, ninety-six well round bottom plates (BD Falcon) were coated with 1 ug/ml of LFA-1 MAb or PBS as a control and stored up to 1 week at 4°C. On the day of the assay, plates were warmed to room temperature and 2 × 10^5^ PBMC were added to each well. After incubation at 37°C for 24 hours, the plates were centrifuged at 1700 rpm for 7 minutes to pellet the cells and the supernatant was removed and stored at −20°C prior to use in subsequent assays.

### HIV replication

To test the direct effect of LFA-1 MAb on HIV-1 replication, the antibody was incubated with virus then a viral replication assay was performed. One hundred TCID_50_ of each virus stock (HIV-SF162, HIV-IIB, HIV-AC225) was incubated for 4 hours at 37°C with serial 10-fold dilutions of LFA-1 MAb. This mixture was then used to infect CD8-depleted PBMC from a single HIV-1 negative donor that had been activated for 3 days with 1.25 ug/ml of PHA in RPMI supplemented with 50U IL-2. Infected cells were maintained at 37°C for 7 days. The degree of viral replication was determined by measuring the concentration of p24 in the supernatant using an ELISA, as per the manufacturers instructions (HIV-1 p24 ELISA, Zeptometrix).

To test the effect of induced soluble factors on HIV-1 replication, a viral replication assay was performed in the presence of supernatant from PBMC treated with LFA-1 MAb or PBS, as described above. PHA activated CD8-depleted PBMC from a single HIV-1 negative donor were resuspended in supernatants diluted 1:2 with culture media, then infected with 10 TCID50 of HIV-SF162 and incubated at 37°C for 7 days. The concentration of p24 was then measured using an ELISA as per the manufacturers instructions (HIV-1 p24 ELISA, Zeptometrix).

### Chemokines

Several assays were used in an attempt to determine the identity of the soluble factor. We started by testing for the presence of cytokines and chemokines that are known to have anti-HIV activity. The concentrations of MIP1-alpha, MIP1-beta, and RANTES were determined using a multiplex bead based ELISA system (Flowcytomix, Bender). Twenty-five microliters of supernatant from PBMC cultures incubated with either LFA-1 MAb or PBS (see above) were mixed with antibody-labeled beads, and biotin-conjugated secondary antibodies. After 2-hours of incubation, the beads were pelleted by centrifugation and incubated with PE-Streptavidin for 1 hour. The beads were then washed and resuspended in assay buffer containing 1% formaldehyde to ensure the samples were not infectious. Preliminary experiments verified that the use of formaldehyde did not interfere with the measurement of the analytes. The fluorescent signal from the beads was detected using a BD LSR-II flow cytometer. The resulting data was analyzed using the Flowcytomix software provided by the manufacturer. The concentration of each chemokine was determined by comparison to a standard curve generated from recombinant chemokines provided by the manufacturer.

An ELISA was performed to determine the concentration of alpha-defensins 1, 2 and 3 (Human HNP 1–3, Hycult Biotech) and Interferon-alpha (Human IFN-alpha pan ELISA kit, MAbtech). One hundred microliters of supernatant from PBMC cultures incubated with LFA-1 MAb or PBS were diluted 1:2 in dilution buffer and the assay was performed in duplicate according to each manufacturer’s instructions. The captured analytes were detected using a biotinylated secondary detection antibody followed by Streptavidin-peroxidase, and developed using a TMB substrate. Serial dilutions of the standards provided by each manufacturer were run in duplicate to create a standard curve from which the concentration of alpha defensins or IFN-alpha was calculated.

To broaden our search for the identity of the soluble factor, we utilized an antibody array (G2000 Human Cytokine Array, RayBioTech). This assay is similar to a standard sandwich ELISA, but it is performed on a glass slide and utilizes a fluorescent readout. This array can detect 174 different analytes including common cytokines, chemokines, and growth factors. A full list of the analytes can be found in Additional file 1. Supernatant from PBMC cultures incubated with either LFA-1 MAb or PBS (see above) was sent to RayBiotech for testing. Since the cell culture media we used in this assay contained serum, which includes many of the analytes being measured, a media only control was also tested. The mean signal intensity of LFA-1 MAb treated supernatants was compared to the mean signal intensity of PBS treated supernatants after subtracting out the signal intensity of the media control. A Wilcoxon sign rank test was used to determine whether the mean signal intensity was higher in LFA-1 MAb treated supernatants.

A follow-up experiment was performed to determine the concentration of Epidermal Growth Factor (EGF) in the LFA-1 MAb and PBS treated supernatants. In this quantitative ELISA, 100ul of supernatant was diluted 1:2 in dilution buffer and the assay was performed in duplicate as indicated by the manufacturer (EGF Human ELISA kit, MAbtech). Duplicate wells of serial dilutions the EGF standard were run in parallel with the samples to generate a standard curve from which the concentration of EGF was calculated.

## Abbreviations

LFA: Lymphocyte funtion-associated antigen; HIV: Human immunodeficiency virus; ELISA: Enzyme linked immunosorbent assay; CTL: Cytotoxic T lympocyte; MAb: Monocolonal antibody; PBMC: Peripheral blood mononuclear cells; PHA: Phytohemaglutinin; CFSE: Carboxyfluorescein succinimidyl ester; PBS: Phosphate buffered saline; MIP: Macrophage inflammatory protein; RANTES: Regulated and normal T cell expressed and secreted; HNP: Human neutrophil protein; IFN: Interferon; EGF: Epidermal growth factor; IL: Interleukin; SDF-1: Stromal cell-derived factor 1; MDC: Macrophage derived chemokines; LIF: Leukemia inhibitory factor; MCP: Monocyte chemoattractant protein; TGF-beta: Transforming growth factor beta; CAF: CD8 antiviral factor.

## Competing interests

This study was funded by Cytodyn. The study design, subject recruitment, and experiments were conceived of and carried out by members of the study team without input from the company.

## Authors’ contributions

JR participated in the design and coordination of the study, performed viral infection experiments, performed the statistical analysis, and drafted the manuscript. GM carried out viral infection experiments and the immunoassays. LJ carried out immunoassays and flow cytometry experiments. SB coordinated the recruitment and examination of study subjects. ER participated in the design and coordination of the study and edited the manuscript. All authors reviewed and approved the final manuscript.

## Supplementary Material

Additional file 1: Table S1 RLU = Relative Light Units. Data shown are the mean signal intensity for each analyte of PBS and Cytolin treated supernatants after subtracting out the signal intensity of the media control. *Unless otherwise indicated, the mean signal intensity in Cytolin treated supernatants was not significantly higher than in control treated supernatants. Click here for file
