# Supplementary material for: A monoclonal antibody against lymphocyte function-associated antigen-1 decreases HIV-1 replication by inducing the secretion of an antiviral soluble factor
Source: Virol J. 2013 Apr 18;10:120. doi: 10.1186/1743-422X-10-120 (PMC3648404; doi:10.1186/1743-422X-10-120)
Supplement: Additional file 1: Table S1 — RLU = Relative Light Units. Data shown are the mean signal intensity for each analyte of PBS and Cytolin treated supernatants after subtracting out the signal intensity of the media control. *Unless otherwise indicated, the mean signal intensity in Cytolin treated supernatants was not significantly higher than in control treated supernatants. [file 1743-422X-10-120-S1.pdf]

Supplemental Table 1

ANALYTE	MEAN PBS (RLU)	MEAN CYTOLIN (RLU)	SIGNIFICANCE*
**Acrp30**	-2421	-1537	
**Activin A**	43	44	
**AgRP**	51	46	
**ALCAM**	1012	562	
**Amphiregulin**	80	50	
**Angiogenin**	-3086	-1294	
**Angiopoietin-2**	27	41	
**Axl**	29	28	
**b-NGF**	-22	-40	
**B7-1(CD80)**	173	146	
**BDNF**	115	79	
**bFGF**	-16	-34	
**BLC**	20	35	
**BMP-4**	-1	10	
**BMP-5**	78	86	
**BMP-6**	15	9	
**BMP-7**	-6	-10	
**BTC**	7	35	
**Cardiotrophin-1**	9	-14	
**CCL-28**	60	83	
**CD14**	3785	5536	
**CK beta 8-1**	7	7	
**CNTF**	25	18	
**CTACK**	241	259	
**CXCL- 16**	450	902	
**DR6 (TNFRSF21)**	96	187	
**Dtk**	-96	-125	
**E-Selectin**	236	112	
**EGF**	1230	2241	p=0.03
**EGF-R**	-535	-655	
**ENA-78**	33000	34068	
**Endoglin**	179	35	
**Eotaxin**	10	20	
**Eotaxin-2**	8400	8510	
**Eotaxin-3**	-18	12	
**ErbB3**	210	165	
**Fas Ligand**	53	106	
**Fas/TNFRSF6**	-159	-78	
**FGF-4**	50	27	
**FGF-6**	97	63	
**FGF-7**	30	37	
**FGF-9**	28	60	
**Flt-3 Ligand**	67	72	
**Fractalkine**	20	27	
**GCP-2**	28	30	
**GCSF**	208	253	

**GDNF**	-8	-13		
**GITR**	30	-3		
**GITR-Ligand**	4	0		
**GM-CSF**	6	13		
**GRO**	55167	49098		
**GRO-alpha**	495	320		
**HCC-4**	32	36		
**HGF**	11	6		
**I-309**	-4	-2		
**I-TAC**	9	-1		
**ICAM-1**	-2342	-3768		
**ICAM-2**	-1303	-1235		
**ICAM-3**	1	1		
**IFN-gamma**	68	2		
**IGF-I**	77	84		
**IGF-I SR**	45	-8		
**IGF-II**	4567	2041		
**IGFBP-1**	455	64		
**IGFBP-2**	400	790		
**IGFBP-3**	-329	-174		
**IGFBP-4**	0	-2		
**IGFBP-6**	273	-10		
**IL-1 R II**	51	29		
**IL-1 R4/ST2**	57	28		
**IL-1 RI**	18	2		
**IL-10**	61	90		
**IL-10 Rbeta**	58	74		
**IL-11**	48	19		
**IL-12 p40**	132	121		
**IL-12 p70**	-10	-25		
**IL-13**	63	75		
**IL-13 Ralpha2**	46	42		
**IL-15**	68	94		
**IL-16**	22	34		
**IL-17**	67	24		
**IL-18 BPalpha**	96	124		
**IL-18 Rbeta**	-70	-15		
**IL-1alpha**	24	-3		
**IL-1beta**	148	143		
**IL-1ra**	64	20		
**IL-2**	34	42		
**IL-2 Ralpha**	154	157		
**IL-2 Rapha**	37	8		
**IL-2 Rbeta**	14	14		
**IL-2 Rgamma**	78	146		
**IL-21R**	15	12		
**IL-3**	26	12		
**IL-4**	20	13		
**IL-5**	100	90		

**IL-5 Ralpha**	10	8		
**IL-6**	536	272		
**IL-6 R**	286	554		
**IL-7**	49	6		
**IL-8**	37445	46598		
**IL-9**	94	27		
**IP-10**	66	5		
**L-Selectin**	-4475	-3857		
**LAP**	4071	4795		
**Leptin**	93	111		
**Leptin R**	122	62		
**LIF**	94	91		
**LIGHT**	20	31		
**Lymphotactin**	19	18		
**M-CSF**	28	25		
**M-CSF R**	84	51		
**MCP-1**	17531	19432		
**MCP-2**	24	24		
**MCP-3**	17575	20702		
**MCP-4**	7	20		
**MDC**	242	151		
**MIF**	1943	1973		
**MIG**	30	21		
**MIP-1-delta**	7	8		
**MIP-1alpha**	7692	8473		
**MIP-1beta**	32216	33829		
**MIP-3-alpha**	9	15		
**MIP-3beta**	9	14		
**MMP-1**	130	114		
**MMP-13**	-10	-28		
**MMP-9**	526	323		
**MPIF-1**	12	28		
**MSP-alpha**	-5831	-4476		
**NAP-2**	6570	6863		
**NGF R**	130	167		
**NT-3**	51	13		
**NT-4**	38	35		
**Oncostatin M**	177	173		
**Osteoprotegerin**	48	148		
**PARC**	39	67		
**PDGF AA**	2035	899		
**PDGF Ralpha**	2	-1		
**PDGF Rbeta**	70	19		
**PDGF-AB**	339	210		
**PDGF-BB**	8877	7715		
**PECAM-1**	66	33		
**PIGF**	9	1		
**Prolactin**	4	8		
**RANTES**	33710	40335		

**SCF**	6	30	
**SCF R**	99	280	
**SDF-1**	31	40	
**SDF-1beta**	30	40	
**sgp130**	202	192	
**Siglec-5**	5839	6156	
**sTNF RII**	1748	2951	
**sTNF-RI**	64	33	
**TARC**	57	58	
**TECK**	16	17	
**TGF beta2**	72	21	
**TGF-alpha**	-10	-20	
**TGF-beta 1**	118	145	
**TGF-beta 3**	-11	1	
**Thrombopoietin**	-12	0	
**Tie-1**	-29	-4	
**Tie-2**	7	15	
**TIMP-1**	4240	2995	
**TIMP-2**	-2524	-5141	
**TIMP-4**	226	171	
**TNF-alpha**	57	79	
**TNF-beta**	73	57	
**TRAIL R3**	7	4	
**TRAIL R4**	4	3	
**uPAR**	10844	10554	
**VE-Cadherin**	84	25	
**VEGF**	21	31	
**VEGF R2**	60	29	
**VEGF R3**	19	9	
**VEGF-D**	33	40	

RLU = Relative Light Units

Data shown are the mean signal intensity for each analyte of PBS and Cytolin treated supernatants after subtracting out the signal intensity of the media control.

*Unless otherwise indicated, the mean signal intensity in Cytolin treated supernatants was not significantly higher than in control treated supernatants.
